# Prevalence, determinants, and outcomes of SARS‐COV‐2 infection among cancer patients. A population‐based study in northern Italy

**DOI:** 10.1002/cam4.4271

**Published:** 2021-09-22

**Authors:** Diego Serraino, Antonella Zucchetto, Luigino Dal Maso, Stefania Del Zotto, Francesca Taboga, Elena Clagnan, Lucia Fratino, Francesca Tosolini, Ivana Burba

**Affiliations:** ^1^ Unit of Cancer Epidemiology Centro di Riferimento Oncologico di Aviano (CRO) IRCCS Aviano Italy; ^2^ Friuli Venezia Giulia Regional Health Coordination Agency Udine Italy; ^3^ Unit of Medical Oncology and Immunesuppression Centro di Riferimento Oncologico di Aviano (CRO) IRCCS Aviano Italy; ^4^ General Directorate Centro di Riferimento Oncologico di Aviano (CRO) IRCCS Aviano Italy

**Keywords:** cancer patients, Italy, mortality, risk factors, SARS‐COV‐2

## Abstract

**Background:**

It is well established that cancer patients infected with SARS‐CoV‐2 are at particularly elevated risk of adverse outcomes, but the comparison of SARS‐CoV‐2 infection risk between cancer patients and cancer‐free individuals has been poorly investigated on a population‐basis.

**Methods:**

A population‐based study was thus conducted in Friuli Venezia Giulia region, northeastern Italy, to estimate prevalence and determinants of SARS‐CoV‐2 infection among cancer patients, as compared to cancer‐free individuals, and to evaluate adverse outcomes of SARS‐CoV‐2 infection. The study included 263,042 individuals tested for SARS‐CoV‐2 in February–December 2020 with cancer history retrieved through the regional cancer registry. Odds ratios (ORs) of SARS‐CoV‐2 positivity, with corresponding 95% confidence intervals (CIs), were calculated using multivariable logistic regression models, adjusted for sex and age. Hazard ratios (HRs) adjusted for sex and age for intensive care unit (ICU) admission and all‐cause death were estimated using Cox models.

**Results:**

Among 26,394 cancer patients tested for SARS‐CoV‐2, the prevalence of infection was 11.7% versus 16.2% among 236,648 cancer‐free individuals, with a corresponding OR = 0.59 (95% CI: 0.57–0.62). The prevalence was much higher (29% in both groups) during the second pandemic wave (October–December 2020). Among cancer patients, age ≥80 years and cancer diagnosis ≥13 months before SARS‐CoV‐2 testing were the major risk factors of infection. Among 3098 infected cancer patients, the fatality rate was 17.4% versus 15.8% among 23,296 negative ones (HR = 1.63, 95% CI: 1.49–1.78), and versus 5.0% among 38,268 infected cancer‐free individuals (HR = 1.23, 95% CI: 1.12–1.36). No significant differences emerged when considering ICU admission risk.

**Conclusion:**

Albeit cancer patients reported reduced SARS‐CoV‐2 infection risk, those infected showed higher mortality than uninfected ones and infected cancer‐free population. Study findings claim for continuing to protect cancer patients from SARS‐CoV‐2, without reducing the level of oncologic care.

## Graphical abstract

SARS‐CoV‐2 infection risk among cancer patients as compared to cancer‐free individuals has been poorly investigated on a population‐basis. This population‐based study found 40% lower odd of SARS‐CoV‐2 positivity among tested cancer patients than cancer‐free individuals. Nonetheless, SARS‐CoV‐2‐positive cancer patients reported higher risk of death, both as compared to negative ones or to positive cancer‐free population. Study findings claim for continuing to protect cancer patients from SARS‐CoV‐2 infection, without reducing the level of oncologic care.

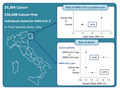

## 1 INTRODUCTION

Biologic (e.g., immune depression related to anticancer treatments) and non‐biologic factors (e.g., most people with cancer needs to interface with health institutions) have been hypothesized to expose cancer patients at higher risk of severe acute respiratory syndrome 2 coronavirus (SARS‐CoV‐2) infection and, as a consequence, at higher risk of COVID‐19 illness than correspondent uninfected cancer patients.^1^, ^2^, ^3^, ^4^

The prevalence of SARS‐CoV‐2 infection among cancer patients showed large variations in estimates, largely dependent on study design, place, and time of investigation. SARS‐CoV‐2 infection was documented among 0.7% of 59,989 Italian cancer patients undergoing active antitumor treatment according to a retrospective nation‐wide study.^5^ Among 4789 cancer patients tested for SARS‐CoV‐2 in northern Italy (i.e., the first area affected by COVID‐19 in Europe), 723 (15.1%) turned out to have acquired SARS‐CoV‐2 infection,^1^ whereas, a study from Reggio Emilia province found that out of 27,386 cancer patients, 1527 were tested for SARS‐CoV‐2, and 447 (1.6%, 29.5% of those tested) resulted positive.^6^ Other investigations have indirectly assessed the prevalence of SARS‐CoV‐2 infection in cancer patients. For instance, 6%–7% of 5700 COVID‐19 patients hospitalized in New York turned out to be cancer patients, a proportion higher than expected according to cancer prevalence in the general population.^7^

Several investigations have highlighted that cancer patients infected with SARS‐CoV‐2 are at particularly elevated risk of adverse outcomes.^1^, ^3^, ^6^, ^8^, ^9^, ^10^, ^11^ A pooled analysis of 52 studies published as of July 2020 projected a high probability of death for infected cancer patients, with a case fatality rate of 25.6%.^8^ Findings from an international cohort study identified factors associated with an increased 30‐day all‐cause mortality, including old age, male sex, smoking, number of comorbidities, and active cancer.^9^

Conversely, few large investigations evaluated the risk of COVID‐19 and adverse outcomes among cancer patients as compared to cancer‐free individuals in the general population, reporting heterogeneous results.^3^, ^6^

The aims of this population‐based study were to estimate prevalence of, and factors associated with, SARS‐CoV‐2 positivity among people with cancer who underwent molecular swab test in northeastern Italy, making comparisons with the cancer‐free population. Furthermore, the study aimed to assess adverse outcomes of infection, that is, admission in intensive care unit (ICU) and death, among SARS‐CoV‐2‐positive cancer patients, both as compared to negative ones and to positive cancer‐free individuals.

## 2 METHODS

A retrospective population‐based study was carried out on all resident individuals of the Friuli Venezia Giulia (FVG) region (northeastern Italy, 1,210,000 inhabitants) who underwent molecular SARS‐CoV‐2 testing between February and December 2020.

We took advantage from real‐world data derived from the regional health information system warehouse, which covers the whole resident population and includes the totality of health‐related databases available––for administrative purposes––in both public and affiliated private facilities. De‐identified data can be cross‐linked through an encrypted code, which is changed every 6 months in order to guarantee patient anonymity. The study was approved by the Bioethics Committee of the Veneto Regional Authority (protocol No. 245343/2020).

For the aims of this investigation, the following data sources were used: microbiological laboratory records; hospital discharge database; Johns Hopkins Adjusted Clinical Group (ACG) system^®^ database; cancer registry; diabetes registry; and municipal registries.

The study population consisted of 263,042 individuals who underwent a SARS‐CoV‐2 nasopharyngeal swab test analyzed by means of real‐time reverse transcription‐polymerase chain reaction (RT‐PCR), between 26 February and 31 December 2020. Tested people were classified as positive or negative to SARS‐CoV‐2 infection based on test results. For individuals with multiple tests, those with all negative results were considered as SARS‐CoV‐2 negative at the date of the first test, while those with at least one positive result were considered as SARS‐CoV‐2 positive at the date of the first positive test.

To assess the cancer history, the above data were linked with the regional cancer registry that records incident cancer cases since 1995 and was updated up to December 2017. The cancer registry data includes all malignant neoplasms (International Classification of Diseases 10th version, ICD‐10, codes: C00‐C96) and benign, in situ, and uncertain behavior neoplasms of bladder (ICD‐10: D30.3, D09.0, D41.4). For the years 2018–2020, information on cancer history was obtained from hospital discharges and the pathological laboratory records. For patients with multiple cancers, the most recent cancer diagnosis was considered, before or up to 30 days from SARS‐CoV‐2 testing (i.e., concurrent diagnosis). The hospital discharge database (updated on 31 December 2021) was used to assess admissions to ICUs, while information on vital status was obtained from municipal registries (updated to 6 February 2021). The Johns Hopkins ACG system^®^ database (based on the following data sources: hospital discharge, pharmaceutical prescriptions, access to emergency departments, and prescription charge exemptions), was used to assess the presence of comorbidities up to the year 2019.^12^ The presence of chronic conditions was evaluated including the following Medical Conditions (MCs) items: congestive heart failure, diabetes, hypertension, ischemic heart disease, obstructive pulmonary disease; the presence of cardiovascular and respiratory diseases was derived from the corresponding Major Expanded Diagnosis Clusters (MEDCs). The presence of diabetes mellitus was assessed through the regional diabetes registry updated to 2019.

Among all individuals tested for SARS‐CoV‐2, the odds ratios (ORs) of a positive result, with the corresponding 95% confidence intervals (CIs), were calculated for cancer patients as compared to cancer‐free individuals (reference category), overall and in strata of selected characteristics, using multivariable logistic regression models, adjusted for sex and age class (<40, 40–59, 60–69, 70–79, and ≥80 years).^13^ Within the group of cancer patients tested for SARS‐CoV‐2, risk factors for a positive result were also evaluated, by calculating the ORs, and corresponding 95% CI, adjusted for sex and age class.

The risks of admission to ICU and the risk of death for any cause among SARS‐CoV‐2‐positive cancer patients were evaluated both in comparison with SARS‐CoV‐2‐negative cancer patients and with SARS‐CoV‐2‐positive cancer‐free individuals, overall and in strata of patient characteristics. Hazard ratios (HRs) of the event of interest, and the corresponding 95% CI, were estimated using proportional Cox model adjusted for sex and age class.^14^ Time at risk of ICU admission was computed from the date of SARS‐CoV‐2 test up to the date of first ICU admission, death, or 31 December 2020, whichever came first. Time at risk of death was computed from the date of SARS‐CoV‐2 test up to the date of death, or 6 February 2021, whichever came first.

## 3 RESULTS

As of December 2020, 263,042 residents in the study area (19.5% of the whole population) underwent at least one molecular swab test for SARS‐CoV‐2. A history of cancer diagnosed before––or at time of––testing was documented in 26,394 of them (10.0%). Table 1 illustrates the distribution of tested individuals according to cancer history, SARS‐CoV‐2 test result, and selected characteristics. People tested for SARS‐CoV‐2 with a history of cancer had a lower prevalence of SARS‐CoV‐2 infection (11.7%) than cancer‐free individuals (16.2%), with a corresponding 41% risk reduction (sex and age adjusted OR = 0.59, 95% CI: 0.57–0.62). Statistically significant reduced risks of testing positive to SARS‐CoV‐2 for cancer patients were documented in all the examined subgroups. However, during the second wave of the pandemic in Italy (i.e., October–December 2020) the risk reduction was less marked (OR = 0.74, 95% CI: 0.71–0.78).

**TABLE 1:** Distribution of 263,042 tested individuals for SARS‐CoV‐2 according to cancer history^a^ and result of SARS‐CoV‐2 test (February–December 2020)^b^

	Cancer patients (*n* = 26,394)			Cancer‐free individuals (*n* = 236,648)		OR (95% CI) of positive test^c^, ^d^
	SARS‐CoV‐2 test			SARS‐CoV‐2 test		
	Positive	Negative	OR (95% CI) of positive test^c^	Positive	Negative	As compared to cancer‐free individuals
	*N* (%)	*N*	Within cancer patients	*N* (%)	*N*	
All	3098 (11.7)	23,296	—	38,268 (16.2)	198,380	0.59 (0.57–0.62)
Sex						
Men	1452 (11.3)	11,369	1^f^	17,876 (16.6)	89,792	0.57 (0.54–0.60)
Women	1646 (12.1)	11,927	1.09 (1.01–1.17)	20,392 (15.8)	108,588	0.63 (0.59–0.66)
Age (years)						
<40	91 (10.7)	758	1.02 (0.80–1.28)	12,177 (13.1)	80,611	0.80 (0.64–0.99)
40–59	575 (11.1)	4601	1.05 (0.92–1.19)	13,709 (17.8)	63,497	0.59 (0.54–0.64)
60–69	526 (10.5)	4473	1^f^	4189 (17.6)	19,621	0.55 (0.50–0.61)
70–79	814 (10.6)	6833	1.02 (0.91–1.14)	3480 (17.8)	16,062	0.55 (0.51–0.59)
≥80	1092 (14.1)	6631	1.40 (1.25–1.57)	4713 (20.2)	18,589	0.67 (0.62–0.72)
Chronic conditions^e^						
No	980 (11.0)	7905	1^f^	25,975 (15.5)	142,139	0.56 (0.53–0.61)
Yes	2118 (12.1)	15,391	1.04 (0.95–1.14)	12,293 (17.9)	56,241	0.61 (0.58–0.64)
1	933 (11.8)	6984	1.02 (0.91–1.13)	5628 (18.1)	25,484	0.59 (0.54–0.63)
≥2	657 (13.0)	4409	1.12 (0.99–1.26)	2509 (17.5)	11,870	0.71 (0.64–0.77)
Unknown	528 (11.7)	3998	1.02 (0.90–1.15)	4156 (18.0)	18,887	0.58 (0.52–0.64)
Cardiovascular diseases						
No	1693 (11.2)	13,418	1^f^	31,063 (15.9)	164,906	0.56 (0.53–0.59)
Yes	1405 (12.5)	9878	1.07 (0.99–1.16)	7205 (17.7)	33,474	0.65 (0.61–0.69)
Respiratory diseases						
No	2673 (11.8)	20,020	1^f^	36,282 (16.2)	187,687	0.59 (0.57–0.62)
Yes	425 (11.5)	3276	0.94 (0.84–1.05)	1986 (15.7)	10,693	0.62 (0.56–0.70)
Diabetes mellitus						
No	2448 (11.4)	19,044	1^f^	35,136 (16.0)	184,320	0.58 (0.55–0.60)
Yes	650 (13.3)	4252	1.17 (1.06–1.29)	3132 (18.2)	14,060	0.67 (0.61–0.74)
Period of SARS‐CoV−2 test						
Feb–May	294 (3.6)	7842	1^f^	2811 (5.6)	47,018	0.48 (0.42–0.55)
Jun–Sep	48 (0.5)	8879	0.14 (0.10–0.19)	1122 (1.7)	66,371	0.45 (0.34–0.61)
Oct–Dec	2756 (29.5)	6575	11.22 (9.91–12.72)	34,335 (28.8)	84,991	0.74 (0.71–0.78)
Time since cancer diagnosis (months)						
<13	466 (5.9)	7404	1^f^	—	—	0.28 (0.26–0.31)
13–24	317 (10.3)	2763	1.83 (1.57–2.12)	—	—	0.52 (0.46–0.58)
25–60	597 (13.2)	3934	2.41 (2.12–2.73)	—	—	0.69 (0.63–0.75)
>60	1718 (15.7)	9195	2.88 (2.58–3.20)	—	—	0.83 (0.79–0.88)

^a^ Cancer diagnosed before SARS‐CoV‐2 testing or no later than 30 days after SARS‐CoV‐2 testing.

^b^ Friuli Venezia Giulia Region, Italy, 26 February–31 December 2020.

^c^ Odds ratio (OR) and corresponding 95% confidence interval (CI) of a positive versus negative test result, estimated using logistic regression model adjusted by sex and age class.

^d^ Cancer‐free individuals as reference category.

^e^ It includes the following Medical Conditions of the ACG^®^ system: congestive heart failure, diabetes, hypertension, ischemic heart disease, and obstructive pulmonary disease.

^f^ Reference category.

Among 26,394 cancer patients, women were at a slightly higher risk of testing positive than men (OR = 1.09, 95% CI: 1.01–1.17), patients aged 80 years or older were at significantly increased risk than younger ones (OR = 1.40, 95% CI: 1.25–1.57) (Table 1). Thirteen percent of cancer patients with two or more chronic conditions were infected with SARS‐CoV‐2, as compared to 11% among cancer patients without chronic conditions (OR = 1.12, 95% CI: 0.99–1.26). Diabetes mellitus was significantly associated with increased risk (OR = 1.17, 95% CI: 1.06–1.29). The detection of SARS‐CoV‐2 infection significantly increased with increasing time elapsed between cancer diagnosis and testing, from 5.9% among those tested within 12 months from cancer diagnosis to 15.7% among those tested more than 60 months after cancer (OR = 2.88, 95% CI: 2.58–3.20). The proportion of people with cancer infected with SARS‐CoV‐2 greatly increased from 3.6% of those tested during the first wave (i.e., February–May 2020) of the pandemic to 0.5% in June–September, and 29.5% during the second wave (i.e., October–December) (OR = 11.22, 95% CI: 9.91–12.72), mirroring the pattern found in cancer‐free individuals.

**FIGURE 1:**
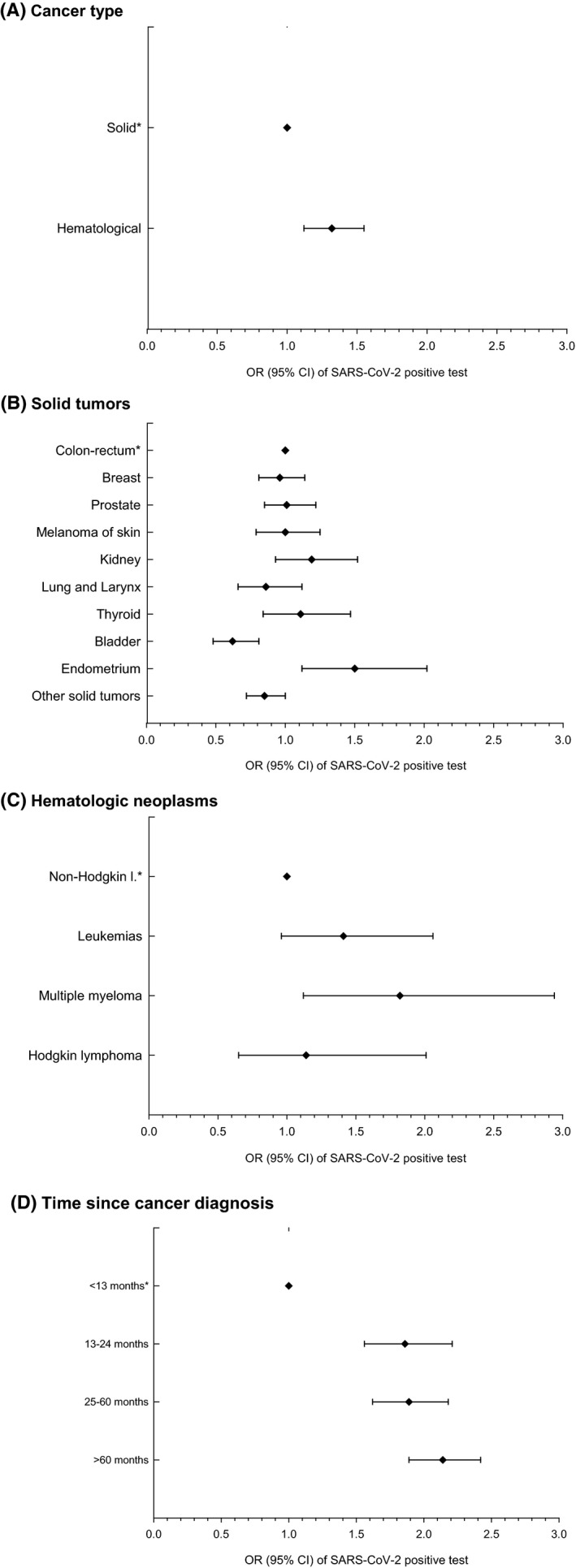
OR^a^ of SARS‐CoV‐2 positive test during the second pandemic wave (October–December 2020) among 9331 cancer patients^b^. Notes: ^a^Odds Ratios (OR) and corresponding 95% confidence intervals, estimated using logistic regression models adjusted for age and sex. ^b^According to type of neoplasm (A–C) and time from cancer diagnosis (D). Friuli Venezia Giulia Region, Italy, 1 October–31 December 2020. ^*^Reference Category

Given such remarkable difference between the two pandemic waves in the FVG region and the large prevalence of infection during the second wave, a subgroup analysis was carried out with focus on the 9331 cancer patients tested in October–December 2020 (Table 2). SARS‐CoV‐2 infection was documented in 29.1% of 8597 patients with solid tumors and in 34.5% of 734 patients with hematological malignancies. Wide intra‐group variations were noted, from 21.3% (bladder cancer) to 38.7% (women with endometrial cancer) in individuals with solid tumors; and from 29.6% (Hodgkin lymphoma) to 46.5% (multiple myeloma) in those with hematological malignancies. As compared with 119,326 cancer‐free individuals, patients with solid tumors were at lower risk of SARS‐CoV‐2 positivity (OR = 0.72, 95% CI: 0.68–0.76), whereas, no difference emerged for those with hematological malignancies (OR = 1.00). For most cancer sites, statistically significant risk reductions emerged. Only people with multiple myeloma turned out to be at increased risk of infection (OR = 1.55, 95% CI: 1.01–2.37). Within the group of cancer patients, those with hematological malignancies were at significantly higher risk of testing positive than those with solid tumors (OR = 1.32, 95% CI: 1.12–1.55) (Table 2 and Figure 1A). In comparison with colorectal cancer (i.e., the most frequent type in both sexes combined), patients with bladder cancer had significantly reduced risk (OR = 0.62, 95% CI: 0.48–0.81), whereas women with endometrial cancer were at a significantly increased risk (OR = 1.50, 95% CI: 1.12–2.02) (Figure 1B). Among hematological cancer patients, those with multiple myeloma showed a 1.82‐fold more elevated risk of infection (95% CI: 1.12–2.95) than non‐Hodgkin lymphoma patients (Figure 1C). As compared to patients with a recent diagnosis (i.e., <13 months), all those tested 13 or more months after cancer diagnosis were at significantly increased risk of infection (OR = 1.86, 95% CI: 1.56–2.21, for 13–24 months, OR = 1.89, 95% CI: 1.62–2.18, for 25–60 months, OR = 2.14, 95% CI: 1.89–2.42, for >60 months) (Figure 1D).

**TABLE 2:** Distribution of 9331 cancer patients^a^ by SARS‐CoV‐2 test result and type of neoplasm, during the second pandemic wave (October–December 2020)^b^

Type of neoplasm	SARS‐CoV‐2 test			
	Positive	Negative	OR (95% CI) of positive test^c^	
	*N* (%)	*N*	Within cancer patients (*n *= 9331)	As compared to cancer‐free individuals^d^ (*n* = 119,326)
Solid tumors	2503 (29.1)	6094	1^e^	0.72 (0.68–0.76)
Breast	593 (29.0)	1451	0.96 (0.81–1.14)	0.71 (0.64–0.78)
Prostate	411 (30.9)	920	1.01 (0.85–1.22)	0.76 (0.67–0.85)
Colon–rectum	352 (30.9)	787	1^e^	0.74 (0.65–0.84)
Melanoma of skin	159 (29.4)	381	1.00 (0.79–1.25)	0.77 (0.64–0.93)
Kidney	130 (33.9)	254	1.19 (0.93–1.52)	0.89 (0.72–1.10)
Lung and Larynx	101 (26.9)	275	0.86 (0.66–1.12)	0.64 (0.51–0.81)
Thyroid	99 (30.8)	222	1.11 (0.84–1.47)	0.91 (0.72–1.16)
Bladder	94 (21.3)	348	0.62 (0.48–0.81)	0.46 (0.37–0.58)
Endometrium	94 (38.7)	149	1.50 (1.12–2.02)	1.09 (0.84–1.42)
Other solid tumors	470 (26.5)	1307	0.85 (0.72–1.00)	0.65 (0.58–0.72)
Hematological neoplasms	253 (34.5)	481	1.32 (1.12–1.55)	1.00 (0.86–1.17)
Non‐Hodgkin lymphomas	118 (31.7)	254	1^e^	0.83 (0.67–1.04)
Leukemias	69 (36.7)	119	1.41 (0.96–2.06)	1.14 (0.84–1.53)
Multiple myeloma	40 (46.5)	46	1.82 (1.12–2.95)	1.55 (1.01–2.37)
Hodgkin lymphoma	26 (29.6)	62	1.14 (0.65–2.01)	1.04 (0.65–1.65)

^a^ In case of multiple tumors, the most recent diagnosis has been selected.

^b^ Friuli Venezia Giulia Region, Italy, 1 October–31 December 2020.

^c^ Odds ratio (OR) and corresponding 95% confidence interval (CI) of a positive versus negative test result estimated using logistic regression model adjusted by sex and age class.

^d^ Cancer‐free individuals as reference category.

^e^ Reference category.

The clinical impact of SARS‐CoV‐2 infection among the whole group of 26,394 cancer patients was assessed considering ICU admission and all‐cause death among those positive as compared to those negative to SARS‐CoV‐2 (Table 3). Overall, 3.0% of 3098 positive and 4.1% of 23,296 negative cancer patients were admitted to an ICU, with no difference in the corresponding hazard (HR = 0.99). Statistically significant elevated risk of ICU admission emerged for cancer patients aged 70–79 years (HR = 1.44, 95% CI: 1.06–1.97), for those with respiratory diseases (HR = 1.80, 95% CI: 1.18–2.73), and for those diagnosed with cancer more than 60 months before testing (HR = 1.38, 95% CI: 1.02–1.86). Men with prostate cancer (HR = 1.94, 95% CI: 1.26–2.98) and hematological cancer patients positive for SARS‐CoV‐2 (HR = 1.95, 95% CI: 1.02–3.75) were at significantly elevated risk of admission in ICU than negative ones.

**TABLE 3:** Distribution of 26,394 cancer patients^a^ tested for SARS‐CoV‐2 by test result, ICU admission, and death (February–December 2020)^b^

	SARS‐CoV‐2 Positive			SARS‐CoV‐2 Negative			HR of ICU^c^, ^d^ (95% CI)	HR of death^c^, ^e^ (95% CI)
	Total	ICU	Death	Total	ICU	Death		
	*N*	*N* (%)	*N* (%)	*N*	*N* (%)	*N* (%)		
All cancer patients	3098	93 (3.0)	540 (17.4)	23,296	960 (4.1)	3678 (15.8)	0.99 (0.80–1.22)	1.63 (1.49–1.78)
Sex								
Men	1452	68 (4.7)	318 (21.9)	11,369	596 (5.2)	2098 (18.5)	1.19 (0.93–1.54)	1.83 (1.62–2.06)
Women	1646	25 (1.5)	222 (13.5)	11,927	364 (3.1)	1580 (13.3)	0.67 (0.45–1.01)	1.39 (1.21–1.60)
Age (years)								
<70	1192	23 (1.9)	55 (4.6)	9832	322 (3.3)	774 (7.9)	0.80 (0.52–1.23)	1.02 (0.77–1.34)
70–79	814	46 (5.7)	129 (15.9)	6833	354 (5.2)	1056 (15.5)	1.44 (1.06–1.97)^g^	1.57 (1.30–1.89)^g^
≥80	1092	24 (2.2)	356 (32.6)	6631	284 (4.3)	1848 (27.9)	0.74 (0.48–1.12)^g^	1.73 (1.54–1.94)^g^
Chronic conditions^f^								
No	980	13 (1.3)	86 (8.8)	7905	215 (2.7)	728 (9.2)	0.65 (0.37–1.15)	1.41 (1.12–1.77)
Yes	2118	80 (3.8)	454 (21.4)	15,391	745 (4.8)	2950 (19.2)	1.07 (0.85–1.35)	1.67 (1.51–1.85)
Cardiovascular diseases								
No	1693	37 (2.2)	207 (12.2)	13,418	479 (3.6)	1645 (12.3)	0.81 (0.58–1.13)	1.44 (1.24–1.67)
Yes	1405	56 (4.0)	333 (23.7)	9878	481 (4.9)	2033 (20.6)	1.15 (0.87–1.52)	1.76 (1.56–1.98)
Respiratory diseases								
No	2673	67 (2.5)	398 (14.9)	20,020	783 (3.9)	2715 (13.6)	0.84 (0.65–1.08)	1.58 (1.42–1.76)
Yes	425	26 (6.1)	142 (33.4)	3276	177 (5.4)	963 (29.4)	1.80 (1.18–2.73)	1.84 (1.53–2.20)
Diabetes mellitus								
No	2448	60 (2.5)	363 (14.8)	19,044	716 (3.8)	2737 (14.4)	0.89 (0.68–1.15)	1.53 (1.37–1.72)
Yes	650	33 (5.1)	177 (27.2)	4252	244 (5.7)	941 (22.1)	1.25 (0.86–1.80)	1.84 (1.56–2.17)
Period of testing								
Feb–Sep	342	22 (6.4)	112 (32.8)	16,721	794 (4.8)	3211 (19.2)	1.43 (0.93–2.18)	1.54 (1.28–1.87)
Oct–Dec	2756	71 (2.6)	428 (15.5)	6575	166 (2.5)	467 (7.1)	1.13 (0.85–1.49)	2.34 (2.05–2.67)
Time from cancer diagnosis (months)								
<13	466	13 (2.8)	119 (25.5)	7404	504 (6.8)	1597 (21.6)	0.56 (0.32–0.98)	1.78 (1.47–2.15)
13–60	914	27 (3.0)	138 (15.1)	6697	194 (2.9)	1037 (15.5)	1.45 (0.96–2.17)	1.51 (1.26–1.81)
>60	1718	53 (3.1)	283 (16.5)	9195	262 (2.9)	1044 (11.4)	1.38 (1.02–1.86)	2.12 (1.85–2.42)
Type of cancer								
Solid	2818	82 (2.9)	486 (17.3)	21,227	892 (4.2)	3368 (15.9)	0.93 (0.74–1.16)	1.59 (1.45–1.76)
Breast	673	8 (1.2)	70 (10.4)	4660	69 (1.5)	335 (7.2)	0.95 (0.45–1.98)	1.68 (1.29–2.18)
Prostate	460	27 (5.9)	108 (23.5)	2781	102 (3.7)	376 (13.5)	1.94 (1.26–2.98)	2.57 (2.06–3.21)
Colon‐rectum	407	17 (4.2)	98 (24.1)	2740	206 (7.5)	455 (16.6)	0.71 (0.43–1.17)	2.17 (1.73–2.71)
Hematological	280	11 (3.9)	54 (19.3)	2069	68 (3.3)	310 (15.0)	1.95 (1.02–3.75)	2.04 (1.52–2.74)
Non‐Hodgkin lymphomas	129	6 (4.7)	21 (16.3)	1023	41 (4.0)	152 (14.9)	1.73 (0.72–4.17)^g^	1.65 (1.04–2.63)^g^
Leukemias	77	1 (1.3)	20 (26.0)	529	14 (2.7)	98 (18.5)	0.73 (0.09–5.63)^g^	2.26 (1.38–3.71)^g^

^a^ Cancer diagnosed before SARS‐CoV‐2 testing or later than 30 days after SARS‐CoV‐2 testing.

^b^ Friuli Venezia Giulia Region, Italy, 26 February–31 December 2020.

^c^ Hazard Ratio (HR) and corresponding 95% confidence interval (CI) of event for SARS‐CoV‐2‐positive versus SARS‐CoV‐2‐negative cancer patients, estimated using Cox models adjusted for sex and age class.

^d^ Follow‐up from SARS‐CoV‐2 test up to ICU admission, death, or 31 December 2020, whichever came first.

^e^ Follow‐up from SARS‐CoV‐2 test up to death, or 6 February 2021, whichever came first.

^f^ It includes the following Medical Conditions of the ACG^®^ system: congestive heart failure, diabetes, hypertension, ischemic heart disease, and obstructive pulmonary disease.

^g^ Cox model adjusted for sex and years of age as a continuous term.

Among positive cancer patients, the percentage of deaths was 17.4% as compared to 15.8% among negative ones, with a corresponding 1.6‐fold increased risk of death (HR = 1.63, 95% CI: 1.49–1.78) (Table 3). This increased risk was documented in all strata of patient characteristics––aside than in those younger than 70 years––but with some differences across strata. The HRs were higher among males (HR = 1.83, 95% CI: 1.62–2.06, vs. HR = 1.39 in females), increased with age (HR = 1.73, 95% CI: 1.54–1.94 in those aged ≥80 years), were higher among cancer patients with chronic conditions (HR = 1.67, 95% CI: 1.51–1.85, vs. HR = 1.41 in those without), with cardiovascular diseases (HR = 1.76, 95% CI: 1.56–1.98, vs. HR = 1.44 in those without), with respiratory diseases (HR = 1.84, 95% CI: 1.53–2.20, vs. HR = 1.58 in those without), with diabetes mellitus (HR = 1.84, 95% CI: 1.56–2.17, vs. HR = 1.53 in those without), and tested during the second pandemic wave (HR = 2.34, 95% CI: 2.05–2.67, vs. HR = 1.54 in the first wave). Particularly high hazards of death emerged for SARS‐CoV‐2‐positive patients diagnosed with cancer more than 60 months before testing (HR = 2.12, 95% CI: 1.85–2.42). When considering cancer type, patients with prostate cancer (HR = 2.57, 95% CI: 2.06–3.21), colorectal cancer (HR = 2.17, 95% CI: 1.73–2.71), or a hematological neoplasm (HR = 2.04, 95% CI: 1.52–2.74 above all leukemia, HR = 2.26), reported the highest risks of death. SARS‐CoV‐2 infection exerted a strong negative impact on survival of cancer patients, in particular within the first 30 days following the result of a positive test, when 448 (14.5%) of positive cancer patients deceased as compared to 1394 (6.0%) of negative ones, with a corresponding HR = 2.40 (95% CI: 2.15–2.67). These findings were consistent in all strata of patient characteristics (data not shown).

Among individuals positive to SARS‐CoV‐2, the fatality rate was 17.4% among 3098 with cancer history and 5.0% among 38,268 cancer‐free ones, with a corresponding sex and age adjusted 1.2‐fold higher risk of death (95% CI: 1.12–1.36) (Table 4). Conversely, no statistically significant differences emerged with regard to the risk of ICU admission, aside from those with respiratory diseases reporting a 2‐fold increased risk (HR = 2.04, 95% CI: 1.26–3.31). The excess risk of death emerged in all the considered subgroups of patients, except those older than 80 years and those diagnosed with cancer more than 60 months before testing. Particularly elevated excess death risks were documented for younger cancer patients (e.g., those aged <70 years, HR = 4.01, 95% CI: 2.93–5.49), without history of chronic conditions (HR = 1.55, 95% CI: 1.22–1.96), with hematological tumors (HR = 1.79, 95% CI: 1.36–2.34)––in particular, leukemia (HR = 2.23, 95% CI: 1.44–3.47), and with more recent cancer diagnosis (e.g., <13 months before test, HR = 1.93, 95% CI: 1.61–2.33).

**TABLE 4:** Distribution of 41,366 SARS‐CoV‐2‐positive individuals by cancer history,^a^ ICU admission, and death (February–December 2020)^b^

	Cancer patients			Cancer‐free individuals			HR of ICU^c^, ^d^ (95% CI)	HR of death^c^, ^e^ (95% CI)
	Total	ICU	Deaths	Total	ICU	Deaths		
	*N*	*N* (%)	*N* (%)	*N*	*N* (%)	*N* (%)		
All SARS‐COV‐2‐positive individuals	3098	93 (3.0)	540 (17.4)	38,268	502 (1.3)	1914 (5.0)	1.09 (0.87–1.37)	1.23 (1.12–1.36)
Sex								
Men	1452	68 (4.7)	318 (21.9)	17,876	370 (2.1)	869 (4.9)	1.04 (0.79–1.35)	1.26 (1.10–1.43)
Women	1646	25 (1.5)	222 (13.5)	20,392	132 (0.7)	1045 (5.1)	1.25 (0.81–1.92)	1.24 (1.07–1.44)
Age (years)								
<70	1192	23 (1.9)	55 (4.6)	30,075	236 (0.8)	153 (0.5)	1.36 (0.88–2.10)	4.01 (2.93–5.49)
70–79	814	46 (5.7)	129 (15.9)	3480	173 (5.0)	370 (10.6)	1.09 (0.79–1.52)^g^	1.40 (1.14–1.71)^g^
≥80	1092	24 (2.2)	356 (32.6)	4713	93 (2.0)	1391 (29.5)	0.87 (0.56–1.37)^g^	1.09 (0.97–1.23)^g^
Chronic conditions^f^								
No	980	13 (1.3)	86 (8.8)	25,975	139 (0.5)	362 (1.4)	1.12 (0.63–2.01)	1.55 (1.22–1.96)
Yes	2118	80 (3.8)	454 (21.4)	12,293	363 (3.0)	1552 (12.6)	1.04 (0.81–1.33)	1.17 (1.05–1.30)
Cardiovascular diseases								
No	1693	37 (2.2)	207 (12.2)	31,063	288 (0.9)	751 (2.4)	0.98 (0.69–1.39)	1.39 (1.19–1.62)
Yes	1405	56 (4.0)	333 (23.7)	7205	214 (3.0)	1163 (16.1)	1.16 (0.86–1.57)	1.13 (1.00–1.28)
Respiratory diseases								
No	2673	67 (2.5)	398 (14.9)	36,282	452 (1.2)	1470 (4.1)	0.93 (0.71–1.20)	1.20 (1.07–1.34)
Yes	425	26 (6.1)	142 (33.4)	1986	50 (2.5)	444 (22.4)	2.04 (1.26–3.31)	1.26 (1.04–1.53)
Diabetes mellitus								
No	2448	60 (2.5)	363 (14.8)	35,136	376 (1.1)	1406 (4.0)	1.02 (0.77–1.35)	1.22 (1.09–1.37)
Yes	650	33 (5.1)	177 (27.2)	3132	126 (4.0)	508 (16.2)	1.19 (0.80–1.76)	1.23 (1.04–1.47)
Period of testing								
Feb–Sep	342	22 (6.4)	112 (32.8)	3933	109 (2.8)	377 (9.6)	1.26 (0.78–2.03)	1.36 (1.09–1.68)
Oct–Dec	2756	71 (2.6)	428 (15.5)	34,335	393 (1.1)	1537 (4.5)	1.05 (0.81–1.35)	1.20 (1.08–1.34)
Time from cancer diagnosis (months)								
<13	466	13 (2.8)	119 (25.5)	—	—	—	0.90 (0.52–1.56)	1.93 (1.61–2.33)
13–60	914	27 (3.0)	138 (15.1)	—	—	—	1.10 (0.74–1.62)	1.31 (1.10–1.56)
>60	1718	53 (3.1)	283 (16.5)	—	—	—	1.12 (0.84–1.50)	1.02 (0.90–1.16)
Type of cancer								
Solid	2818	82 (2.9)	486 (17.3)	—	—	—	1.04 (0.82–1.32)	1.19 (1.07–1.31)
Breast	673	8 (1.2)	70 (10.4)	—	—	—	0.98 (0.48–2.00)	0.92 (0.72–1.17)
Prostate	460	27 (5.9)	108 (23.5)	—	—	—	1.15 (0.77–1.71)	1.04 (0.85–1.27)
Colon‐rectum	407	17 (4.2)	98 (24.1)	—	—	—	1.40 (0.86–2.27)	1.34 (1.10–1.65)
Hematological	280	11 (3.9)	54 (19.3)	—	—	—	1.50 (0.83–2.73)	1.79 (1.36–2.34)
Non‐Hodgkin lymphomas	129	6 (4.7)	21 (16.3)	—	—	—	1.71 (0.76–3.82)	1.32 (0.86–2.03)
Leukemias	77	1 (1.3)	20 (26.0)	—	—	—	0.45 (0.06–3.20)	2.23 (1.44–3.47)

^a^ Cancer diagnosed before SARS‐CoV‐2 testing or later than 30 days after SARS‐CoV‐2 testing.

^b^ Friuli Venezia Giulia Region, 26 February–31 December 2020.

^c^ Hazard Ratio (HR) and corresponding 95% confidence interval (CI) of event for cancer patients versus cancer‐free individuals, estimated using Cox models adjusted for sex and age class.

^d^ Follow‐up from SARS‐CoV‐2 test up to ICU admission, death, or 31 December 2021, whichever came first.

^e^ Follow‐up from SARS‐CoV‐2 test up to death, or 6 February 2021, whichever came first.

^f^ It includes the following Medical Conditions of the ACG^®^ system: congestive heart failure, diabetes, hypertension, ischemic heart disease, and obstructive pulmonary disease.

^g^ Cox model adjusted for sex and years of age as a continuous term.

The study findings did not materially change when risk estimates (ORs and HRs) were further adjusted for the presence or the number of chronic conditions, or for the presence of diabetes, respiratory, and cardiovascular diseases (data not shown).

## 4 DISCUSSION

The findings from this population‐based study showed that, between February and December 2020 in northeastern Italy, 26,394 cancer patients who were tested for SARS‐CoV‐2 by RT‐PCR had about 40% lower risk of resulting positive, as compared to 236,648 cancer‐free individuals in the general population who were tested in the same geographical area. On the other side, the study found that cancer patients who were positive to SARS‐CoV‐2 reported higher risks of death, as compared to both negative cancer patients (HR = 1.6) and, to a lesser extent, to cancer‐free individuals who were positive to SARS‐CoV‐2 (HR = 1.2). Among cancer patients, female sex, age older than 80 years, presence of diabetes mellitus, a longer cancer history, and, above all, the second wave of the pandemic in Italy (i.e., October–December 2020, during which 29.5% of tested cancer patients turned out to be positive) were factors associated with SARS‐CoV‐2 infection. Findings from a large population‐based study conducted in the United States,^3^ including more than 2.5 million cancer patients, of whom 1200 (0.05%) with COVID‐19 reported a higher risk of COVID‐19 among cancer patients, in particular among those with a recent cancer diagnosis (OR = 7.1). The authors suggested that patients with a recent cancer were more exposed to SARS‐CoV‐2 infection due to high levels of health care contacts. In this study, conversely, we found that a less recent cancer diagnosis was associated with greater risk of testing positive to SARS‐CoV‐2. Another population‐based study conducted in Northern Italy during the first pandemic wave^6^ reported an elevated probability of being tested for SARS‐CoV‐2, especially in those with a cancer diagnosis made in the previous 2 years. Of note, differently from our study, the two above mentioned studies included not only individuals tested for SARS‐CoV‐2, but they considered all those not tested as uninfected. Such heterogeneous results are probably due to non‐biologic factors, in particular to different approaches for access to the local health care system. In line with the results of other investigations, biologic factors, conversely, are likely to explain the increased risk of infection documented among patients with hematological neoplasms during the second pandemic wave, as compared to those with solid tumors.^3^, ^15^ Patients with hematological malignancies such as leukemias, myelodysplastic syndromes, myeloproliferative neoplasms, lymphomas, and multiple myeloma have usually long‐lasting immunodeficiency due to the malignancy itself, anticancer treatments, or as a consequence of procedures such as hematopoietic stem‐cell transplantation. This makes them particularly susceptible to bacterial and viral infections, such as SARS‐CoV‐2 pneumonia. Consistently with the literature,^16^ our data indicated that patients with hematological neoplasms are a population at high‐risk of poor COVID‐19 outcomes, even in comparison with patients with solid tumors.

Our findings regarding adverse clinical outcomes, particularly death, are in agreement with those of Wang et al.,^3^ showing higher mortality among patients with both cancer and COVID‐19 as compared to both patients with cancer without COVID‐19 and patients with COVID‐19 without cancer. Similar findings emerged also in other investigations.^1^, ^6^, ^8^ The particularly high mortality within 30 days from a positive test was also in agreement with other studies.^9^, ^11^

Among cancer patients, when comparing those positive versus those negative to SARS‐CoV‐2, we found that higher mortality risks emerged for males, older ones, those with chronic diseases and diabetes, in line with the findings of other studies conducted among cancer patients^2^, ^9^, ^11^, ^17^, ^18^, ^19^, ^20^ but also in line with risk factors associated with COVID‐19 mortality in the general population.^19^, ^21^

Conversely, when comparing SARS‐CoV‐2‐positive individuals with cancer history to those without cancer, our findings highlighted that cancer patients were at higher risk of death when they were younger, without cardiovascular diseases or chronic conditions, and with a more recent cancer diagnosis. No differences in mortality emerged according to sex, presence or absence of respiratory diseases or diabetes, or the period of testing. These results are in line with those reported by Mangone et al.^6^

Reasons for being tested for SARS‐CoV‐2 need to be considered with regard to study limitations. These included chiefly the presence of COVID‐19 illness symptoms, the close contacts with SARS‐CoV‐2‐positive individuals, and screening for SARS‐CoV‐2 infection before hospital admission or during hospital stay. It is possible that tested cancer patients included a larger proportion of people who were under active cancer treatment for whom molecular swab test was mandatory (i.e., those admitted to hospitals for chemotherapy or surgical procedures). Due to absence or the very low number of observed adverse outcomes in some sub‐groups of patients (e.g., selected cancer types), our study had not sufficient statistical power to evaluate the risk of event––in particular ICU admission––in all subgroups.

A major strength of this study is the whole coverage of the resident population in the study area, thanks to the availability of a centralized health system data warehouse. This allowed to include all the RT‐PCR test for SARS‐CoV‐2 performed in the Friuli Venezia Giulia region during the study period in public facilities. Molecular swab tests performed in private health facilities and rapid antigen tests were not available, but they were unusual in 2020 in the study area. Another study strength was the use of data from a population‐based cancer registry with a long history and high quality standards in terms of completeness and accuracy of collected data (accredited to the International Association of Research or Cancer and the Italian Association of Cancer Registries).^22^ We are aware that data derived from health system databases can suffer of a reduced quality and completeness of information due to their administrative nature (i.e., they are not collected for clinical or research purposes). For instance, the information on the presence and the number of chronic conditions were derived from the ACG system^®^ database and not from patient's anamnesis, thus we cannot exclude underestimation and misreporting of comorbidities. In addition, some information is not available in the regional health system data warehouse (e.g., smoking habits, body mass index, and type and phase of cancer treatment).

In conclusion, this investigation showed that, albeit the lower risk of testing positive to SARS‐CoV‐2 of cancer patients in Italy, those who were infected suffered of a higher risk of death.

Particular attention should be taken in order to further protect oncologic patients against COVID‐19, while continuing to assure them an adequate access to medical care. In order to reduce the risk of SARS‐CoV‐2 infection among cancer patients and to guarantee the best continuity of care, especially among the elderly, practical recommendations have been made by Italian oncologists.^23^ These measures aim to: (1) favor social distancing and (2) reduce immunodeficiency and iatrogenic treatment‐related events that increase the risk of infections. Among the others, the recommendations included: allowing presence of a single caregiver for a limited time; promoting telephone/telematics triage of disease‐free cancer patients and patients on oral agents treatment; choosing oral targeted therapy over intravenous agents when multiple treatment options are available; employing regimens with a longer interval or prolonging cycle length for disease‐active patients receiving intravenous agents; and avoiding preoperative chemotherapy with high risk of neutropenia for resectable cancer patients.

## CONFLICT OF INTEREST

None.

## ETHICS STATEMENT

The study was approved by the Bioethics Committee of the Veneto Regional Authority (protocol No. 245343/2020).

## INFORMED CONSENT

This epidemiological retrospective study did not imply any direct or indirect intervention on the population considered. Therefore, once the study protocol has been approved by the Bioethics Committee the informed consent is not a requirement.

## Data Availability

Researchers who would like to access individual data should present their request together with a study protocol to the Unit of Cancer Epidemiology, Centro di Riferimento Oncologico di Aviano IRCCS (epidemiology@cro.it).
